# Natural Anti-Microbials for Enhanced Microbial Safety and Shelf-Life of Processed Packaged Meat

**DOI:** 10.3390/foods10071598

**Published:** 2021-07-09

**Authors:** Angelos Papadochristopoulos, Joseph P. Kerry, Narelle Fegan, Catherine M. Burgess, Geraldine Duffy

**Affiliations:** 1 Teagasc Food Research Centre, Ashtown, D15 DY05 Dublin, Ireland; Angelos.Papadochristopoulos@teagasc.ie (A.P.); geraldine.duffy@teagasc.ie (G.D.); 2 Food Packaging Group, School of Food & Nutritional Sciences, University College Cork (UCC), T12 K8AF Cork, Ireland; joe.kerry@ucc.ie; 3 Agriculture and Food, Commonwealth Scientific and Industrial Research Organization (CSIRO), Coopers Plains, Brisbane, QLD 4108, Australia; Narelle.Fegan@csiro.au

**Keywords:** natural anti-microbials, meat products, preservation, anti-microbial packaging, essential oils, plant extracts, flavonoids, organic acids, bacteriocins, nanoparticles

## Abstract

Microbial food contamination is a major concern for consumers and food industries. Consumers desire nutritious, safe and “clean label” products, free of synthetic preservatives and food industries and food scientists try to meet their demands by finding natural effective alternatives for food preservation. One of the alternatives to synthetic preservatives is the use of natural anti-microbial agents in the food products and/or in the packaging materials. Meat and processed meat products are characteristic examples of products that are highly perishable; hence natural anti-microbials can be used for extending their shelf-life and enhancing their safety. Despite several examples of the successful application of natural anti-microbial agents in meat products reported in research studies, their commercial use remains limited. This review objective is to present an extensive overview of recent research in the field of natural anti-microbials, covering essential oils, plant extracts, flavonoids, animal-derived compounds, organic acids, bacteriocins and nanoparticles. The anti-microbial mode of action of the agents, in situ studies involving meat products, regulations and, limitations for usage and future perspectives are described. The review concludes that naturally derived anti-microbials can potentially support the meat industry to provide “clean label”, nutritious and safe meat products for consumers.

## 1. Introduction

Microbial food contamination is a global concern for consumers and food industries and leads to a reduction of product shelf-life and higher risk of foodborne illnesses [1]. Meat is a highly perishable food commodity and this is attributable to its energy-dense nutritional composition which is readily metabolized by microorganisms (fatty acids, amino acids, water-soluble proteins etc.), its high-water content and approximately neutral pH make it an ideal substrate for microbial growth [2]. Contamination of meat occurs on the muscle surface and maybe due to the slaughter animals themselves, processing environment and workers, whilst further processing of the meat into comminuted meat products can lead to cross contamination, and growth of the microbial community may occur post processing and during storage and distribution [1,3,4].

Traditional food preservation methods include chemical, biological and physical methods such as drying, thermal processing, irradiation, freezing, refrigeration, fermentation, modified atmosphere packaging, addition of anti-microbial agents, salting, etc. All these can be used in order to control microbial growth, maintain the functional and quality characteristics of fresh meat, generate novel and unique product characteristics for processed meats and extend the shelf-life of all muscle-based foods [4,5]. However, as indicated many of these approaches are unsuited for application to fresh meat [1]. In the field of fresh meat preservation, the methods feasible for application are based on temperature and moisture control, and, through use of inhibitory processes, such as packaging, high hydrostatic pressure (HPP), etc., often combined with temperature and moisture control employing a hurdle-led approach [6].

Adding anti-microbial agents directly into the product, or more recently by indirect application via the packaging material, are growing areas of interest as potentially acceptable measures for providing enhanced meat preservation. These anti-microbial compounds can be synthetic, or natural in origin [7], and their application may not just deliver preservation effects, but also support food pathogen control in fresh meat products. Additionally, this also raises issues around the legalities of incorporating or applying additives to fresh meat, as it is legally defined and retailed.

Furthermore, some health concerns surrounding the use of additives such as salt and nitrate in meat products has highlighted the drive to find reformulation solutions using natural “clean label” ingredients [8,9].

Salt is an important substance for food products, in terms of their safety and sensory properties. However, according to the World Health Organization (WHO), the consumption of salt must be reduced in order to minimize the risk of heart diseases [10]. High amounts of salt can be found in processed foods, such as ready meals and processed meats. Of course, the reduction of salt in these products will lead to the addition of other substances as salt substitutes or the use of novel technologies, in order to maintain sensory properties and ensure product safety [8].

Another food additive, which is used for curing meat products is sodium nitrite/nitrates. Reduction or removal of this preservative is now a critical issue for meat industry, because, in spite of the several functional advantages of nitrite addition in meat products, which include formation of unique curing colours, flavour, antioxidant potential and anti-microbial properties, there are concerns about its reported possible repercussions on human health [9]. According to several studies, nitrosamines and other reaction products formed from nitrite and nitrate are associated with increased risk of cancer [11]. Therefore, the regulations for application of nitrate in meat products are strict [12] and further reduction of current maximum levels is currently being proposed for legislative uptake within the EU, according to a study conducted by the Food Chain Evaluation Consortium for European Commission [13]. Alternatives are therefore required. Substitution of nitrate with plant extracts, marinades, bacteriocins, bacterial strains and high hydrostatic pressure are some of the methods that have been studied extensively [14,15].

The application of anti-microbial agents to fresh whole intact meat can be as anti-microbial sprays or dips [1]. There are some limitations regarding their direct use on the meat surface because neutralization of the active compounds at the point of contact with the meat surface or quick diffusion from the surface into the meat mass may happen [1].

In processed meats anti-microbial agents may be added as liquid or powder during processing; however, inactivation of the active compounds may occur after interaction with the meat components or upon application of temperatures during cooking, thereby leading to a lower anti-microbial efficacy [1].

Another option for controlling growth of microbial populations in meat products is through incorporation of anti-microbial agents into, or onto, the packaging materials. This is a unique approach in potentially controlling microbial populations as packaging signifies the most important of all preservations steps that can be applied to meat products. The goal of packaging fresh meat is to prevent contamination, delay spoilage, minimize weight loss, enable the activity of enzymes for better tenderness and finally ensure, where it is applicable, that the red meat will retain its oxymyoglobin or bright cherry-red colour when the product reaches the shelves at retail or at customer level [16,17]. On the other hand, according to Mondry [18], factors such as lipid oxidation, dehydration, as well as, the loss of colour and aroma should be also considered for the packaging of processed meat products. Meat packaging includes overwrap packaging for short-term refrigerated storage and/or retail display, vacuum packaging, bulk-gas flushing or modified atmosphere packaging (MAP) systems for long-term refrigerated storage. Moreover, new packaging technologies such as active and intelligent packaging have been developed [17]. Active and intelligent packaging, as defined by the EU, are “materials and articles that are intended to extend the shelf-life or to maintain or improve the condition of packaged food; they are designed to deliberately incorporate components that would release or absorb substances into or from the packaged food or the environment surrounding the food.” and “materials and articles which monitor the condition of packaged food or the environment surrounding the food.”, respectively [19].

Anti-microbial food packaging belongs to the category of active packaging and aims to prolong shelf life by extending the lag phase and reducing the growth rate of microorganisms, whilst preserving its quality characteristics and safety [20]. The incorporation of anti-microbial agents into the packaging could be a better option compared with adding them to the meat products. For instance, plant essential oils are mostly highly volatile and as result it is not necessary to put them in contact with the food, which may lead to potential adverse effects on the sensory properties of the product [21]. Moreover, the slow migration of the anti-microbial agents from the packaging to the surface of the product, leading to maintenance of high concentrations of them until the end of the product shelf-life is of paramount importance [1].

Apart from anti-microbial films as packaging material, the use of edible films and coatings has been also observed. Edible films and coatings are made of polysaccharides, proteins and lipids that have film-forming ability and they demonstrate several important properties, such as biodegradability, anti-microbial, water, vapor, oxygen and carbon dioxide barrier properties [1,22]. According to Gennadios et al. [23], some of the benefits of applying edible films and coatings in meat products are:

- Reduction of moisture loss during storage of fresh or frozen meats.
- Prevention of drip loss when the meat is packed in a plastic tray.
- Lipid and myoglobin oxidation are reduced.
- Decrease of microbial population on the surface of coated meats.
- Effective control of volatile flavour loss and foreign odour pick-up.

The anti-microbial agents can be incorporated, coated, immobilized onto packaging materials, added to sachets/pads inside the packaging, whilst there are also polymers that are intrinsically anti-microbial such as chitosan [24]. Heat stable anti-microbial agents can be incorporated into the packaging materials by compression moulding and extrusion, whereas the heat sensitive anti-microbial compounds can be added by non-thermal methods, such as electrospinning, surface coating and solvent compounding [4]. The anti-microbials can also be incorporated in multilayer films. The active compounds are contained in the matrix layer, whereas the barrier layer inhibits their migration toward the outside of the packaging and the inner layer regulates their diffusion [24]. In the case of immobilization, the anti-microbial agents can be immobilized onto the packaging materials, by ionic or covalent bonds. For this method, the presence of functional groups on both sides, the anti-microbial agent and the polymer is needed [24]. Finally, anti-microbial pads are a very common method for meat packaging. They are used in trays to absorb meat exudates and inhibit bacterial propagation [25].

Generally, before the application of any anti-microbial packaging to meat and processed meat products, several factors should be taken into account; the possible interaction between the food components, anti-microbial agents that should be used and the type of polymer film, the microorganisms to be targeted, the concentration that is required, the microbial kill time, the release rate, the mechanism of action and the effect on the organoleptic properties of the product [21,26]. Generally, higher concentrations of anti-microbials, such as essential oils, are required when applied to food products compared to in vitro studies because food products are complex systems and therefore, there are several interactions between the microorganisms, the active compounds, the food components and external factors (e.g., temperature, type of packaging) that affect the anti-microbial efficacy [27]. Of course, higher dosage may affect the organoleptic properties of the food, however combination of plant essential oils at lower concentrations will have a synergistic effect and therefore higher efficacy as anti-microbial agents, with sensory properties still acceptable for consumers [28,29,30].

## 2. Natural Anti-Microbials

Anti-microbial agents used in food products may be synthetic and chemical or natural in origin [7]. There is growing consumer concern about the use of synthetic preservatives in food and they desire “clean label” food products [31]. Hence, there is a growing interest from food industries in developing and using natural preservatives that meet consumer demands, while still retaining a high quality and flavoursome product.

This review focuses on natural anti-microbial agents, and more specifically on essential oils, plant extracts, flavonoids, organic acids and bacteriocins, that have been studied thoroughly and can be used to ensure the safety and extending the shelf-life of meat and processed meat products.

### 2.1. Essential Oils and Plant Extracts

Extensive research has been conducted in recent times in the area of plant essential oils (EO) and phytochemicals, which are a source of various compounds, such as terpenoids and polyphenols, and they are reportedly able to inhibit the growth of a wide range of microorganisms [4]. Most of the plant essential oils are generally recognized as safe (GRAS) by United States Food and Drug Administration (FDA), whilst several compounds found in essential oils are used as flavouring substances in food products in the European Union according to EU legislation [27,31,32,33,34]; hence, they can be potentially used as anti-microbials in food products, providing a more natural “clean label“ image [27]. A summary of several applications of essential oils and plant extracts directly in meat products or in the packaging materials and coatings can be found in Table 1 and Table 2, respectively.

#### 2.1.1. Thyme and Oregano

Two characteristic examples of plant essential oils that exhibit anti-microbial activity are thyme and oregano essential oils, with the major bioactive compounds being thymol and carvacrol, respectively [72,73]. In addition, thymol can also be found in oregano essential oil and other spices, whilst carvacrol has been isolated from other spices, such as thyme, savoury and marjoram [74].

Thymol and carvacrol share the same anti-microbial mode of action. Xu et al. [75] studied their anti-microbial mechanism against *E. coli*. They observed that these two compounds can disintegrate the outer membrane of Gram-negative bacteria, leading to release of lipopolysaccharide, increase of permeability and depolarization of its potential. In addition, they can also interact with the cell membrane in the Gram-positive bacteria. Ultee et al. [76] observed that the interaction in *Bacillus cereus* caused expansion and destabilisation of the membrane, increasing its permeability, whilst the adenosine triphosphate (ATP) was depleted. In general, the result in both types of bacteria is that the membrane completely loses its integrity and leakage of cellular material such as ions, ATP and nucleic acid is observed, leading to cell death [27]. In another study, Ahmad et al. [77] studied the mechanism of thymol and carvacrol against *Candida* strains. It was observed that thymol and carvacrol can block the synthesis of ergosterol, which is responsible for membrane integrity and disrupt the cell membrane by creating pores.

At the transcriptional level, the mode of action of thymol and carvacrol involves the downregulation of genes involved in cell division, motility and virulence and upregulation of genes associated with stress response, such as envelope stress phage shock operon, heat shock proteins, oxidative stress resistance genes, genes encoding efflux pumps and iron transport. The above was observed when *E. coli* O157:H7 was adapted to sublethal concentration of these anti-microbial agents [78]. Another study connected the upregulation of genes encoding chaperones proteins with the exposure to sublethal concentration of thymol as a response of bacteria to stress conditions [79].

Thyme and oregano essential oils, but also thymol and carvacrol, have been used in many research studies as anti-microbial substances in food products or incorporated into packaging materials. Yemis and Candogan [66] and Emiroglu et al. [67] incorporated thyme and oregano essential oils in soy protein edible films and used the films for packaging of fresh beef cuts and ground beef, respectively. The study of Yemis and Candogan [66] showed that edible coatings with thyme and oregano oil had anti-microbial effect against *Listeria monocytogenes*, *E. coli* O157:H7 and *Staphylococcus. aureus*, but with lower efficacy against *L. monocytogenes*. At the same time, beef had acceptable sensory properties at all concentrations of the essential oils used, whilst a significant improvement of the colour in the beef samples packed with the anti-microbial edible film was observed. On the other hand, in the study of Emiroglu et al. [67] the application of anti-microbial films with thyme and oregano essential oils led to a reduced population of *Pseudomonas* spp. and coliform bacteria. However, no significant effect (*p* > 0.05) on *Staphylococcus* spp. and lactic acid bacteria over storage, was observed. This could be attributed to the fact that ground beef matrix is extremely complex [67]. Furthermore, no big differences in the effectiveness were observed between the essential oils used, however the combination of oregano and thyme essential oils and thyme essential oil alone were significantly more effective against *Pseudomonas* spp. compared to oregano oil.

Thyme and oregano essential oils have also been used as anti-microbial additives to sausages. Garcia-Diez et al. [80] studied the effect of adding essential oils of herbs and spices, such as thyme and oregano oil to dry, cured sausages The results showed significant reduction (*p* < 0.05) of *S. aureus*, whilst *Salmonella* spp. and *L. monocytogenes* were not detected, after 21 days of drying. On the other hand, lactic acid bacteria were not affected by the addition of essential oils and they actually increased during the smoking process. Regarding the influence of essential oils on sensory attributes, 50% and 60% of participants in the consumer test claimed that they would consume the sausage with the low concentration (0.005%) of thyme and oregano essential oil, respectively.

The efficacy of thyme and oregano EO against fungi on meat products has been also studied. Demirok Soncu et al. [71] added thyme oil at a concentration of 1% in chitosan coating solution and applied it to dry fermented sausages. The sausages with the anti-microbial coating had significantly (*p* < 0.05) lower levels of moulds and yeasts compared to control samples after 3 months of storage at 4 °C. Moreover, the treatment with the anti-microbial solution led to preservation of the sensory characteristics of the sausages throughout the storage period. Habashy et al. [37] added thyme oil (0.5% and 1%) to minced beef in order to test its antifungal efficacy against *Aspergillus flavus*. Thyme oil (1%) significantly (*p* < 0.05) reduced the population of *A. flavus* during the storage at 4 °C. In another study, oregano EO was used in order to test its efficacy against moulds during the ripening period of sausages [81]. The application of the essential oil led to reduction of moulds on the surface of the sausage. Regarding the physicochemical properties of the sausage, no significant differences were observed, however in terms of textural characteristics, chewiness and hardness were affected by the treatment.

According to Gutierrez et al. [82] the anti-microbial activity of carvacrol and thymol might be greater at low pH, high concentrations of protein and average levels of sugars, hence the application of the above anti-microbials should be ideal for fermented meat products that have a lower pH.

#### 2.1.2. Rosemary

Rosemary essential oil, which is rich in phenolic acids and flavonoids, shows antioxidant and anti-microbial activity [69,83], hence it has been used for many years as a preservative in the food industry [83]. The anti-microbial action of rosemary essential oil is the result of the interaction of its active compounds with the bacterial membrane and its proteins, leading to dysfunction and disintegration of the membrane, whilst changes in genetic material and nutrients, leakage of cellular content, defective transport of electrons and altered composition of the fatty acids in the cell membrane have been observed [83]. In addition, the antifungal mechanism of rosemary essential oil has been studied. da Silva Bomfim et al. [84] reported that the mode of action of rosemary EO against *Fusarium verticillioides* was based on the disruption of the cell wall, leading to loss of cellular content and blocking the production of fumonisins and ergosterol.

A lot of research in the field of applying rosemary essential oil for inhibiting the growth of microorganisms in meats has been conducted. Sirocchi et al. [69,70] incorporated essential oil from rosemary to an innovative three-layer packaging, by spraying the essential oil on the internal high-density polyethylene (HDPE) layer. Chicken and beef were packaged with this film. Reduction of the biogenic amines produced in the chicken during storage and correlation between biogenic amine production and inhibition of the growth of spoilage bacteria was observed. Beef packaged with anti-microbial packaging containing rosemary oil had its shelf life extended from 5–6 days to 15 days when the anti-microbial packaging was combined with high O_2_ conditions (50% O_2_:30% CO_2_:20% N_2_). The sensory properties of the raw and cooked beef were also evaluated in this study. The colour of meat was acceptable for the anti-microbial packaging in every condition studied for the initial part of storage. In terms of odour, the meat packed in the anti-microbial packaging under vacuum conditions was favoured less by the panellists for the first part of the shelf-life study. Regarding the cooked beef, on the third day of storage, most of the participants in the test were able to distinguish the sample that was packed with the anti-microbial packaging. Similar results were also observed on the third day for the raw meat. These observations were attributed to the intensive odour of rosemary oil. In another study, Liu et al. [51] packaged pork steaks in polyethylene (PE) film with rosemary EO. The anti-microbial packaging was not only effective against Gram-positive but also against Gram-negative bacteria, reducing the growth of *Enterobacteriaceae*. The lowest efficacy of this film was observed on *Pseudomonas* spp. At the same time, the species diversity of spoilage microbiota was not significantly affected from the use of rosemary EO in the film. Finally, the sensory properties of the pork were not affected negatively by the anti-microbial film.

Rosemary oil has been also applied to the surface of chicken breast, which was vacuum packed and stored at low temperature [47,48]. The combination of rosemary essential oil and vacuum packaging extended the shelf-life of chicken from 12 to 16 days. In another study [71], dry sausages were treated with chitosan coating solution contained 1% rosemary oil. The treatment inhibited fungal growth on the casings, whilst there were significantly (*p* < 0.05) lower levels of moulds and yeasts in the sausages compared to control samples after 3 months of storage at 4 °C. In terms of sensory properties, the sausages were acceptable until the end of the storage period.

#### 2.1.3. Cinnamon

Cinnamon, a very common spice used worldwide, is rich in components that have anti-microbial activity, with the major one being *trans*-cinnamaldehyde [27,85]. Cinnamon essential oil can damage the bacterial cell membrane structure in both Gram-positive and Gram-negative bacteria. More specifically, disruption of the membrane, increased electric conductivity, increased permeability, leakage of electrolytes, nucleic acids and proteins and membrane depolarisation have been observed [86]. Cinnamon also has antifungal properties. According to Xing et al. [87], the mode of action of cinnamon EO against *F. verticillioides* involves morphological and structural changes, such as loss of integrity and rigidity of the cell wall, leakage of cytoplasmic content, disruption of membrane, destruction of mitochondria and folding of the cell. Those alterations could be attributed to the interference of cinnamaldehyde with the enzymes involved in cell wall synthesis.

Several studies about the mechanism of action of cinnamon essential oil at molecular level have been conducted. Sheng et al. [88] observed that Shiga Toxin Type 2 (Stx2) protein production and the expression of the *stx2* gene were reduced, whereas genes related to oxidative stress were upregulated when *E. coli* O157:H7 was exposed at 0.25 × MIC (Minimum Inhibition Concentration) level of cinnamon oil. In another study by Lin et al. [89], cinnamaldehyde affected the normal ATP metabolism of *E. coli*. Moreover, the protein synthesis was also affected by upregulating some genes that encode the chaperone proteins DnaK and GroEL, which are extremely common chaperone proteins under stress conditions in *E. coli*. Finally, the genes that are associated with flagellar regulation were downregulated, therefore motility was compromised. Similar findings were also observed in the study of Yuan et al. [78] when *E. coli* O157:H7 was adapted to a sublethal concentration of *trans*-cinnamaldehyde. More specifically, downregulation of genes involved in cell division, motility and virulence and upregulation of genes associated with stress response, such as the envelope stress phage shock operon, heat shock proteins, oxidative stress resistance genes, genes encoding efflux pumps and iron transport was observed.

Cinnamon EO has been incorporated into packaging materials with the purpose of creating an active packaging. Wen et al. [53] incorporated cinnamon EO and β-cyclodextrin into polylacticacid (PLA) nanofibers in order to create a nanofilm by electrospinning and they used it for packing pork, which was later stored at 25 °C. The active nanofilm extended the shelf-life of pork by 5 days. Raeisi et al. [54] incorporated cinnamon EO in a sodium alginate coating and they studied its anti-microbial efficacy on chicken meat. The anti-microbial coating significantly (*p* < 0.05) inhibited the growth of all the bacteria tested and exhibited strong antifungal activity during the 15 days of storage at 4 °C. Van Haute et al. [36] added 1% *w*/*w* cinnamon EO in marinade that was used for chicken breast fillets and pork meat and the result was a reduced growth of yeasts and moulds during the storage of meat at 4 °C. A difference in the odour of raw and baked pork between the samples treated with the anti-microbial marinade and the control was also reported.

#### 2.1.4. Garlic

Garlic essential oil is able to inhibit the growth of pathogens and spoilage microorganisms. The major substance in garlic that has anti-microbial properties is allicin. Allicin is very unstable and can be easily decomposed at high temperatures and pressure [61]. However, its breakdown products, such as diallyl sulphide (DAS) and diallyl disulphide (DAD) also possess anti-microbial properties against pathogenic bacteria [90]. The anti-microbial mechanism of allicin is based on inhibition of several thiol-dependent enzymatic systems inside the cell. At low concentrations the inhibition of these enzymes is sufficient to block the virulence of the microorganism, whereas higher concentrations may lead to death of the microorganism [91]. In addition, Feldberg et al. [92] observed that allicin may partially inhibit DNA, protein and RNA synthesis in *Salmonella enterica* ser. Typhimurium. Finally, the mode of action of garlic essential oil against fungi, such as *Candida albicans* has also been studied. Li et al. [93] observed that garlic EO could penetrate the membrane of the cell, but also the membrane of organelles causing destruction of them. At a molecular level, the expression of genes involved in oxidation-reduction processes, pathogenesis and cellular response to drugs and starvation was induced by treatment with garlic oil.

There are few studies about the anti-microbial effect of garlic essential oil incorporated in packaging material. In one study, garlic oil was incorporated into low density polyethylene (LDPE) films and this was used for packing RTE beef loaf [61]. The anti-microbial film reduced the growth rate of *L. monocytogenes* and a 2 log CFU/g difference in the population was observed after 6 days at 4 °C, for the film containing 8% *w*/*w* garlic oil. A lower concentration of garlic oil (2% *w*/*w*) was also sufficient to control the growth of *L. monocytogenes*. Nevertheless, the anti-microbial film had minimal effect on *E. coli*. Generally, Gram-negative bacteria are more resistant to allicin and also the amount of allicin incorporated in the films may not have been sufficient for inhibiting the growth of *E. coli*. No effect on the growth of *Brochothrix thermosphacta* was observed. Adding the garlic essential oil into the product is also an option for extension of shelf-life and several studies have focused on this method. Garlic EO was added to dry cured sausage and a significant reduction (*p* < 0.05) of *Salmonella* spp., *L. monocytogenes* and *S. aureus* was observed after 21 days of drying. On the other hand, no effect on lactic acid bacteria during the smoking and drying process was observed. Moreover, when the low concentration (0.005%) of garlic oil was added, the sausage had the best organoleptic properties among all the essential oils tested [80].

#### 2.1.5. Pepper

Pepper has anti-microbial properties and because of its composition, with many active compounds, it can target several structures in the bacterial cell impacting on survival and resistance [94,95]. Hence, its anti-microbial mode of action is not characterized by only one specific reaction. Nevertheless, the anti-microbial mechanism of action of black pepper EO, was studied in meat-borne *E. coli* by Zhang, Ye et al. [95]. They showed that the anti-microbial mechanism involved the disruption of the cell membrane that led to leakage of electrolytes, ATP, proteins and DNA, eventually causing the death of bacteria. Other mechanisms may include the destruction of enzymatic mechanisms for energy production, metabolism and DNA and RNA maintenance and anti-quorum sensing [94,96].

Pepper essential oil has been used in many studies as a substance in the food but also incorporated in the packaging material for prolonging the shelf-life of the product and inhibiting the growth of microorganisms. In a recent study from Dalvandi et al. [68], chicken fillets were coated with carboxymethyl cellulose (CMC) solution containing black pepper seed extract and vacuum packed. The application of coating combined with the vacuum packaging led to a reduction of the total aerobic mesophilic and psychrotrophic bacteria, by approximately 2.5 log and 3 log CFU/g, respectively, after 16 days of chilled storage compared to control samples. However, the sensory properties of the chicken were affected by the coating. The meat had a pink colour after application of the coating and it was not accepted by the panellists in the study. Zhang et al. [45] studied the effect of black pepper essential oil, at concentrations of 0.1% and 0.5%, on the quality of pork during storage. Significantly (*p* < 0.05) lower populations of *Enterobacteriaceae* and *Pseudomonas* spp. in the treated samples compared to control samples were observed during the storage period of 9 days at 4 °C. On the other hand, there was no effect on *Brochothrix* spp. and lactic acid bacteria; hence, black pepper EO was more effective against Gram-negative bacteria.

#### 2.1.6. Bay

Bay leaf essential oil mainly contains 1,8-cineol and lower amounts of eugenol, acetyl, methyl-eugenol and α-pinene, linalool among others and it has antioxidant and anti-microbial activities [97,98]. Bay EO can disrupt the membrane of the bacterial cell, increase its permeability, modify the proteins of the membrane and as a result disrupt the transport of molecules through the cell membrane [97].

The addition of bay leaf EO to food could yield an extension to the shelf-life of these products. Addition to sausages and ground chicken meat in order to inhibit the growth of bacteria has been tested. Garcia-Diez et al. [80] added it to cured sausage and as a result *Salmonella* spp., *L. monocytogenes* and *S. aureus* were reduced significantly (*p* < 0.05) after 21 days of drying. No impact on the population of lactic acid bacteria was observed. In terms of organoleptic properties, 60% of the potential consumers favoured the sausages with the low concentration (0.005%) of bay EO. In addition to this, Irkin and Esmer [35] applied bay essential oil on ground chicken meat. The meat was packaged using MAP or vacuum packaging to investigate the combined effect of essential oil with packaging. The combination of bay EO with MAP was the most effective treatment in order to inhibit the growth of *L. monocytogenes* and *E. coli* in ground chicken meat and extend the shelf-life of the product.

#### 2.1.7. Clove

Clove is widely used as a spice and its essential oil is a natural preservative and flavouring substance, which possesses anti-microbial and antioxidant properties [99,100]. The main compound in clove EO is eugenol and the anti-microbial activity of clove is attributed mainly to this compound [101]. The mechanism of anti-microbial activity of clove EO, as has been studied in *S. aureus* by Xu, Liu et al. [101], involves the destruction of the cell wall and membrane, resulting in an increase of permeability and leakage of intracellular content. Furthermore, according to the same study, clove essential oil can block normal DNA and protein synthesis, affecting bacterial growth. In addition, according to Kim et al. [102] clove oil and eugenol can provoke the downregulation of motility genes, curli genes, type I fimbriae genes and *ler*-controlled toxin genes in *E. coli* O157:H7. Curli and fimbriae genes are associated with biofilm formation, attachment and effacement phenotype. Finally, the mode of action of clove essential oil on yeasts and filamentous fungi, as was studied by Pinto et al. [103] is based on the inhibition of ergosterol synthesis. Lesions in the cellular membrane were also observed, whilst the germ tube formation by *C. albicans* was partially or totally inhibited.

Clove EO has been incorporated into films and coatings as an anti-microbial agent. Liu, et al. [51] added clove EO in PE film and packaged pork with it. The microbial species targeted in this study had lower populations in the samples packed with the anti-microbial film compared to control samples during the 7 days of storage at 4 °C. In the same study, the species diversity of spoilage bacteria of the meat packed with and without the anti-microbial film was not significantly different. Moreover, the anti-microbial film did not have any negative impact on the organoleptic properties of the meat. Muppalla and colleagues [55] used carboxymethyl cellulose–polyvinyl alcohol (CMC-PVOH) films with clove EO in order to pack ground chicken meat. The active packaging led to an extension of the shelf-life of the ground meat from 4 to 12 days. Moreover, according to a recent study by Mulla et al. [57], the anti-microbial packaging of a chemically modified linear low density polyethylene film coated with clove EO was effective in reducing the population of pathogenic bacteria in minced chicken meat. *L. monocytogenes* and *S.* Typhimurium were undetectable after the fifth day of cold storage, whereas in the control samples the population of them was around 6 log and 7 log CFU/g, respectively, throughout the storage period of 21 days. The encapsulation of essential oil could lead to higher anti-microbial efficacy. Rajaei et al. [56] encapsulated clove oil in chitosan-myristic acid nanogel and they coated beef with it. The nanogel with the encapsulated clove oil was effective against *S.* Enteritidis during chilled storage, whilst its impact on the meat colour was negligible.

Clove EO has been also added to meat in order to test its antifungal properties. Habashy et al. [37] added clove essential oil at concentrations of 0.5% and 1% to minced beef. The results were very promising when the 1% clove oil was applied. The population of *A. flavus* was reduced, reaching undetectable levels after 9 days of storage at 4 °C.

#### 2.1.8. Peppermint

Peppermint essential oil mainly contains menthone, isomenthone and the different isomers of menthol and it demonstrates antioxidant and anti-microbial properties [104]. Yang et al. [105] studied the anti-microbial mechanism of peppermint EO against *E. coli* and they concluded that it could cause damage to the bacterial cell membrane, increase its permeability and block quorum sensing. In addition, Samber et al. [106] studied the mode of action of peppermint EO against *Candida* species. Reduction of ergosterol levels, inhibition of plasma membrane-ATPase leading to acidification inside the cell and eventually cell death was observed. At the molecular level, peppermint essential oil can provoke a general stress response in bacteria. More specifically, when *Campylobacter jejuni* was exposed to a sublethal concentration of peppermint oil, upregulation of genes encoding chaperone proteins, reduced level of expression of the virulence-related genes and limited motility was observed [107].

The incorporation of peppermint EO in packaging materials has been studied in the past. Talebi et al. [59] added peppermint EO into polylactic acid (PLA) with nanocellulose films. Ground beef meat was packaged with those films. The active films were effective against the bacteria tested. Regarding the sensory attributes of the meat, the low concentration (0.5%) of the essential oil had a negative impact on odour and taste, whereas the application of a higher concentration (1%) led to significantly (*p* ≤ 0.05) better results compared to control samples. Furthermore, peppermint EO has been added to food products in order to extend their shelf-life. Smaoui et al. [46] added peppermint EO, at concentration level of 0.25–0.5%, to minced beef meat stored at 4 °C and as a result its shelf life was extended from 7 to 14 days. The sensory properties were also evaluated in this study. Peppermint EO at the level of 0.25% led to an acceptable product for 14 days. On the other hand, the minced meat became unacceptable by the panellists after 7 days when the high concentration (0.5%) of essential oil was used.

#### 2.1.9. Nutmeg

Nutmeg is a very common spice and its essential oil is rich in α-pinene, sabinene, β-pinene, limonene, 4-terpineol and α-terpineol [65]. According to Kiarsi et al. [65], nutmeg essential oil shows higher efficacy against Gram-negative bacteria in contrast to other essential oils from spices and herbs, because of its surface activity and higher affinity for the external membrane of these bacteria, which consists mainly of lipids.

The anti-microbial properties of nutmeg EO has been described in several publications. In a study by Kiarsi et al. [65], beef packed with a nutmeg EO/sage seed mucilage coating had prolonged shelf-life. Moreover, when the coating with 2% nutmeg EO was applied, the limit of 7 log CFU/g for the total viable counts was not reached during the 6 days of storage at 4 °C. Lipid oxidation was retarded and the firmness and the red colour of the meat were preserved. No significant difference in terms of the sensory properties was observed, although the panellists evaluated the colour, odour and overall acceptability of the samples with the anti-microbial coating after 6 days of chilled storage with slightly higher scores. Šojić et al. [42] applied nutmeg EO at two different concentrations (10 ppm and 20 ppm) on cooked sausage and they observed lower populations of total aerobic mesophilic counts compared to the control and no difference or enhanced aroma in terms its sensory characteristics throughout the 60 days of cold storage.

#### 2.1.10. Lemongrass

Lemongrass produces an aromatic essential oil and the principal components present are citral, neral and geranyl acetate [108]. Citral (C_10_H_16_O) is a monoterpenoid aldehyde, a mixture of neral and geranial, with a distinct odour. Aside from lemongrass EO, it can be found in citrus fruits, myrtle trees and basil [109]. Lemongrass EO in general and citral have anti-microbial properties [109,110]; hence, lemongrass oil can be potentially used as a natural food preservative.

Tyagi and Malik [111] studied the damage to the structure of *E. coli* due to the anti-microbial activity of lemongrass. They observed it involves the disruption of the cell membrane and loss of its integrity, leakage of cytoplasmic content and finally impeding cell growth. Furthermore, citral could influence the ATP concentration and membrane hyperpolarization in *Cronobacter sakazakii*, whilst the pH inside the cell was decreased [99]. Lemongrass EO is very effective against *L. monocytogenes*, owing to the disruption of lipid bonds in the cell membrane [112]. According to Hadjilouka et al. [113], lemongrass EO can affect the expression of genes involved in fatty acid biosynthesis, peptidoglycan biosynthesis and virulence-related genes. The virulence genes *hly* and *inlJ* of *L. monocytogenes* were downregulated after exposure to lemongrass oil, whereas upregulation and downregulation of several other genes, depending on the strain of *L. monocytogenes* tested and the concentration used, was also observed.

Lemongrass EO has been incorporated into films and coatings aimed at inhibiting the growth of pathogenic and spoilage bacteria. PLA, which is a biodegradable polymer containing lemongrass EO was used as anti-microbial packaging for pork sausages. The films incorporated with 2% essential oil inhibited the growth of *L. monocytogenes*. The population of the microorganism was approximately 1.5 log CFU/g lower in comparison with the control samples after 12 days storage at 4 °C [64].

#### 2.1.11. Coriander—Linalool

Coriander is a spice with a very distinctive odour and taste. The main active compound found in its essential oil is linalool, whilst α-pinene, γ-terpinene and camphor are some of other constituents of it [114]. Linalool (3,7-dimethyl-octa-1,6-diene-3-ol), is an acyclic monoterpene tertiary alcohol, which is a volatile component found in the essential oils of several plant extracts in addition to coriander EO, such as basil and citrus essential oils [115,116]. Linalool demonstrates several biological activities such as antibacterial, anti-inflammatory and antioxidant [117].

The anti-microbial mechanism of linalool against *P. aeruginosa* was reported by Liu, Cai et al. [118]. Membrane disintegration, increase of membrane permeability, leakage of cellular material, depolarisation of the cell membrane, abnormal cell metabolism and damage in the respiratory chain was observed. The result of all these adverse effects was cell death. Gao et al. [117] studied the anti-microbial mechanism of linalool against *L. monocytogenes* at the transcriptional level. The mechanism involved the upregulation of genes related to peptidoglycan biosynthesis, osmoprotectant genes, genes related to glycine betaine, iron uptake, cation binding, uptake of carbohydrates, ribosomes, RNA degradation and DNA replication. On the other hand, linalool induced downregulation of genes associated with the transport of materials inside and outside the cell. The mechanism of coriander essential oil against *Candida* spp. has also been studied. Freires et al. [119] reported that coriander essential oil was able to bind to membrane ergosterol, causing an increase in permeability and disruption of the membrane leading to cell death.

Coriander oil has been incorporated in packaging materials in order to prolong the shelf-life of food products. Kargozari et al. [58] added coriander oil into an edible coating of sodium alginate and they used it for the packaging of chicken fillets. The populations of aerobic bacteria, psychrotrophic bacteria, yeasts, moulds, lactic acid bacteria, coliforms and *S. aureus* were significantly (*p* < 0.05) lower in the chicken fillets packaged with the anti-microbial coating in comparison with the control samples throughout the 12 days of refrigerated storage. Moreover, the anti-microbial coating exhibited strong antifungal activity by inhibiting the growth of yeast and moulds in chicken fillets packaged with it. There was no significant difference regarding the sensory properties of the chicken when the coating with a low concentration (0.5%) of coriander oil was used. On the other hand, the coating with a higher concentration (1%) had a slightly negative impact on the product, however it was still satisfactory. In addition, the anti-microbial coating minimised lipid oxidation.

Michalczyk et al. [38] applied coriander EO on ground beef which was stored at 0.5 or 6 °C. Limited anti-microbial effect was observed except for *Enterobacteriaceae* that demonstrated a difference of 1–2 log CFU/g in treated samples compared to the control, throughout the storage period. The limited efficacy was attributed to the low concentration of the essential oil used. Regarding the sensory properties of the meat, the addition of the essential oil had no negative impact, whilst in some samples it enhanced them.

#### 2.1.12. Horseradish—Mustard (Allyl-Isothiocyanate (AITC))

Mustard, horseradish, cabbage and wasabi are some of the members of *Brassicaceae* family and the major flavour substance found in them is allyl-isothiocyanate (AITC) [62]. This volatile component has been used as a preservative for many years and it demonstrates anti-microbial activity against a broad range of microorganisms [120]. Moreover, according to Suhr and Nielsen [121], AITC has higher anti-microbial efficiency when used in the vapour rather than the liquid form. Nevertheless, use of it in food products is limited because of its strong odour, which can affect the taste of the food [122]. For this reason, the continuous release of low amounts of AITC is proposed as this might extend the time of treatment, improve its anti-microbial efficacy, while the negative impact of its distinct odour on the organoleptic properties of the product will be decreased [62]. The AITC anti-microbial mode of action involves cell penetration, disintegration of the cell membrane, leakage of intracellular materials and inhibition of enzymes involved into the redox balance and metabolism [123,124].

AITC had been extensively used as an anti-microbial agent in food products and packaging films because of its high efficacy. According to Dias et al. [63], the combination of allyl-isothiocyanate with carbon nanotubes incorporated into a cellulose-based film, at concentrations higher than 28% and 0.02%, respectively, led to reduction of *Salmonella* Choleraesuis, psychrotrophic and mesophilic bacteria in shredded cooked chicken throughout the storage period of 40 days at 4 °C. Changes in colour and lipid oxidation were also minimized. Shin et al. [62] applied a control release of AITC in vapour combined with MAP (30% CO_2_:70% N_2_) on chicken. It was observed that the combination of AITC and MAP could successfully inhibit the growth of *S.* Typhimurium and *L. monocytogenes*. However, a slight discolouration of the chicken was also observed as a side effect from the use of AITC at release rates above 1.2 µg/h.

#### 2.1.13. Cumin

Cumin essential oil contains γ-terpinene, cymene, terpenoids, α-pinene and cuminaldehyde among others [114] and it also possesses anti-microbial and antioxidant properties [59,125,126]. The anti-microbial mechanism of cumin is based on the anti-microbial activity of monoterpenes and mostly of cymene. According to Ultee et al. [76] p-cymene caused expansion of the cytoplasmic membrane of *B. cereus* resulting in leakage of ions.

Many studies have been conducted in order to evaluate the efficacy of cumin EO in packaging materials. Sharafati Chaleshtori et al. [60] added cumin EO in a chitosan-based coating and used it for packing chicken meat. The anti-microbial coating was very effective in inhibiting the growth of mesophilic bacteria, lactic acid bacteria, *Enterobacteriaceae*, moulds and yeasts and prolonging the shelf-life of the chicken meat. In addition to this, the overall acceptability of the product in terms of sensory properties was not significantly (*p* > 0.05) affected when a low concentration (0.5%) was used. In another study, Talebi and colleagues [59] incorporated essential oil from black zira which is a spice similar to cumin into polylactic acid (PLA) with nanocellulose films and they used them in order to pack ground beef meat. The active films showed anti-microbial efficacy and on top of that the odour, colour and taste were approximately equal or enhanced when the ground meat was treated with them.

#### 2.1.14. Turmeric

Turmeric is a rhizomatous shrub and it has anti-microbial and antioxidant properties [127,128]. Its principal bioactive component is curcumin, which is a natural, polyphenolic compound and it also demonstrates anti-microbial activity [129]. According to Tyagi et al. [129], the anti-microbial mode of action of curcumin against *S. aureus* and *E. coli* involves damaging the cell membrane of the bacteria by increasing its permeability, which leads to excessive leakage of cellular components.

Turmeric water extract has been used as an anti-microbial agent in active packaging systems. Dalvandi et al. [68], used a carboxymethyl cellulose (CMC) solution containing turmeric extract in order to coat chicken fillets. The combined application of coating with vacuum packaging extended the shelf-life of chicken breast from 7 to more than 16 days. At the same time, the fillets coated with turmeric extract showed low levels of proteolysis and lipid oxidation, whereas the sensory properties of the chicken were enhanced by the presence of the coating.

#### 2.1.15. Hop

The hop plant, apart from giving a unique bitter taste to beer, has significant anti-microbial and antioxidant properties. The anti-microbial activity of hop is due to α-acids (humulones), β-acids (lupulones) and xanthohumol and it has been demonstrated to be mainly active against Gram-positive bacteria [130]. According to Simpson [131], the anti-microbial mechanism of hop compounds against lactic acid bacteria is due to the presence of β-acids, α-acids, but mainly iso-α-acids. The hop compounds could act as carriers of ions provoking a reduction of pH inside the cell, inhibition of nutrient uptake, leading to starvation and finally the death of the cell.

Hop extract has been used as a preservative. Kramer et al. [41] added hop extract (β-acids) at a concentration of 5000 ppm to meat marinade and pork loins were vacuum packed with this marinade. Inhibition of the growth of *L. monocytogenes*, total aerobic counts and lactic acid bacteria in the meat with the anti-microbial marinade compared to control was observed, during the 2 weeks of cold storage. Regarding the sensory properties of the meat, the distinct bitter taste of hop extracts was not observed in this study. Moreover, according to Kramer et al. [41], the application of hop extracts should be preferably applied in products with low fat and low pH values and at chilled storage in order to have higher efficacy as an anti-microbial agent.

#### 2.1.16. Chrysanthemum

Another essential oil with anti-microbial activity is chrysanthemum essential oil (CHEO), which is extracted from chrysanthemum. Its anti-microbial mode of action involves the disruption of the cell membrane and the increase of membrane permeability, leading to leakage of cellular substances and eventually the death of bacteria [52]. Moreover, Lin, Mao et al. [52] incorporated CHEO in a chitosan-nanofibers film through electrospinning and they used this film for packing beef. This anti-microbial film demonstrated 98% reduction of *L. monocytogenes* population after 24 h at 4 °C, whilst it reduced lipid oxidation.

### 2.2. Flavonoids

Flavonoids are part of plant secondary metabolites and they are widely found in fruits, vegetables, nuts, seeds, stems, flowers, tea, propolis, cocoa and honey and they possess anti-microbial activities among others [132,133]. Most of the plant extracts that are rich in flavonoids are generally recognized as safe (GRAS) by the FDA [134,135,136]. In this part of the review, an overview of research studies focusing on the plant extracts and propolis that naturally contain flavonoids, their anti-microbial activity and their application on meat products will be presented. In Table 3 several research studies are presented where anti-microbial agents rich in flavonoids were applied directly in meat products or incorporated into active packaging.

#### 2.2.1. Grapefruit Seed Extract

Grapefruit seed extract (GFSE) is a natural substance, which is extracted from seeds and pulp of grapefruit, it is rich in flavonoids and other polyphenols, it is effective against a wide spectrum of Gram-positive and Gram-negative bacteria [148,149] and it has antifungal properties. The anti-microbial mode of action of GFSE has been studied in Gram-negative and Gram-positive bacteria by Heggers et al. [150]. Disruption of the cell membrane and leakage of cellular material, leading to death of the cell was reported. Furthermore, the mechanism of action of grapefruit seed extract against *Saccharomyces cerevisiae* has been studied. Cao et al. [151] observed that grapefruit seed extract could provoke apoptosis in *S. cerevisiae*, by destructing the mitochondrial 60 S ribosomal protein L14-A and blocking the conversion of pantothenic acid to coenzyme A (CoA).

Many studies have been conducted by incorporating GFSE in the packaging material in order to enhance the shelf-life of food products. Ha et al. [143] packaged ground beef in low density polyethylene (LDPE) film, in which GFSE was incorporated. The films were made with two different methods: co-extrusion and solution-coating and the latter one showed superior anti-microbial activity by extending the shelf-life of ground beef. In another study, Hong et al. [144] packed pork loins with *Gelidium corneum*–gelatin film which contained GFSE (0.08%). The populations of the *E. coli* O157:H7 and *L. monocytogenes* in the pork loins were significantly (*p* < 0.05) lower compared to control samples after 10 days of storage at 4 °C.

#### 2.2.2. Grape Seed Extract

Grape seed extract (GSE) is a by-product of vinification and grape juice processing and it is rich in flavonoids, such as procyanidins [135]. It is beneficial for human health; however, its use is limited because of its poor solubility in water, degradation at high temperatures and low bioavailability [152]. GSE has significant anti-microbial activity against pathogenic and spoilage bacteria [153]. The anti-microbial mechanism of GSE against bacteria is based on the presence of flavonoids and it includes disruption of the cell membrane, inhibition of enzymes in the membrane and dissipation of the proton motive force [135].

GSE has been used in many studies for innovative active packaging in order to delay oxidation and spoilage. Sogut and Seydim [141] used GSE combined with chitosan in order to create a film for packing chicken breast meat. All the combinations of different concentrations (5–15%) of GSE with chitosan limited the microbial growth and lipid oxidation in chicken fillets during storage at 4 °C and prolonged their shelf-life.

#### 2.2.3. Green Tea Extract

Green tea has several reported health benefits, such as antioxidant, anti-carcinogenic, anti-arteriosclerotic and anti-microbial properties [135]. The main compound found in green tea is catechins, which are mainly responsible for its anti-microbial activity [154]. The antibacterial activity of green tea catechins includes several mechanisms, which operate simultaneously. Some of them are more general, whereas others are specific for certain bacteria. Those mechanisms include inhibition of virulence factors (toxins and biofilm formation), excessive disruption of the cell wall and cell membrane, inhibition of enzymatic activities, oxidative stress, disruption of DNA and RNA metabolism and iron chelation [154].

Green tea extract (GTE), because of its anti-microbial properties, has been widely used in studies in order to extend the shelf-life of food products by being incorporated into active packaging. Hong et al. [144] used *Gelidium corneum*–gelatin films with 2.8% GTE for packaging pork meat. For *E. coli* O157:H7 and *L. monocytogenes*, 1 log CFU/g difference after 10 days of chilled storage between the samples in the active packaging and the control samples was observed. Ozruval et al. added green tea extract to beef burgers by direct addition, incorporation into chitosan edible coating and encapsulation. The hamburger patties that were coated with chitosan-encapsulated GTE solution exhibited the lowest level of mesophilic bacteria and coliforms compared to all the other treatments after 8 days of storage at 4 °C. On the other hand, it was observed that the patties with the above treatment had higher population of yeasts and moulds compared to the control throughout the storage period. It was assumed that the encapsulation prevented the antifungal activity of GTE.

#### 2.2.4. Propolis Extract

Propolis is a natural resinous substance produced by honeybees from collected plant buds [137]. There are several different chemical compounds that have been found in the composition of propolis. However, the composition of it shows a big variation around the world and of course depends on its botanical origin [155]. The main compounds found in propolis belong to the categories of phenolic acids and flavonoids, whilst new phenolic compounds are sometimes discovered [156]. Propolis possesses several biological activities, such as antibacterial, antiviral and antifungal [156,157,158]. The anti-microbial activity of propolis against Gram-positive and Gram-negative bacteria is complex and may be attributed to several different mechanisms, including prevention of cell division, increase of membrane permeability, disruption of membrane potential and ATP production and inhibition of bacterial motility [159,160]. On the other hand, the antifungal activity of propolis can be attributed to its phenolics and flavonoids, which affect the permeability of the cytoplasmic membrane, leading to leakage of the cellular content and finally the death of the cell [158].

Being a good source of flavonoids, propolis is a potential active agent that can be incorporated into films or added to food products. Skowron et al. [137] combined ethanolic extracts of propolis at different concentrations (10% and 20%) with chitosan and this mixture was coated onto polypropylene film (PP). This anti-microbial film was used in packaging of salami. It was observed that the film with propolis and chitosan significantly reduced (*p* < 0.05) the population of *L. monocytogenes* in salami during the 15 days storage. In a study by Vargas-Sánchez et al. [146], propolis extracts at 2% *w*/*w* were added to beef patties. A 19.75% and 27.03% reduction of growth of mesophilic and psychrotrophic bacteria, respectively, was observed after 8 days cold storage for the samples treated with the non-commercial propolis. The commercial propolis extracts also reduced to the same or lesser extent the growth of the bacteria tested. In addition, lipid oxidation was also decreased. Ali et al. [147] added ethanolic extract of propolis (0.6%) to sausages and they reported lower level of proteolytic, lipolytic and yeasts and moulds counts in the treated samples compared to the control during storage at 5 °C, whilst the shelf-life of sausages was prolonged from 12 to 21 days.

The antifungal activity of propolis extract on meat products has also been studied. Ozturk [161] evaluated the activity of ethanolic extract of propolis on the natural microbiota of a fermented sausage. A reduction of 2.05 log CFU/g in the population of yeasts and moulds compared to control after 12 days of ripening was reported. Moreover, the population of Micrococcaceae was significantly (*p* < 0.05) lower in comparison with control throughout the ripening period. On the other hand, propolis extract was not so effective against lactic acid bacteria and aerobic mesophilic bacteria. It was also noted that the treatment did not affect the colour of the sausage.

#### 2.2.5. Cranberry

Cranberry is a fruit and has been used for medicinal purposes historically. The main compounds found in berries, in general, are flavonoids, such as anthocyanins and flavonols [162,163] and these compounds demonstrate antioxidant and anti-microbial activity among others [163,164,165].

Cranberry concentrate is able to provoke disruption of the bacterial cell wall, cell membrane, intracellular matrix and leakage of cytoplasmic content in Gram-negative and Gram-positive bacteria [166]. According to Wu et al. [138] who studied the anti-microbial mechanism of action of cranberry concentrate against *E. coli* O157:H7 at the molecular level, downregulation of genes related to cell membrane biosynthesis was observed. Moreover, genes that were induced during stress conditions, such as acid, starvation and osmotic stress, were also downregulated in the samples treated with the cranberry extract. Hence, the anti-microbial compounds of cranberry concentrate can block the transcription of genes and the synthesis of proteins that are necessary for growth. Diarra et al. [167] studied the effect of cranberry extract against *S. aureus*. The results showed that the cranberry press cake extract affected the transcription of genes involved in the synthesis of peptidoglycan, which is the main component of the cell wall of Gram-positive bacteria.

Cranberry has been studied as a preservative in meat products. Tamkute et al. [139] added cranberry pomace ethanol extract, at level of 2% to pork slurry, burger and cooked ham. Significant (*p* < 0.05) inhibition of growth of *L. monocytogenes* in all of the pork meat products with the cranberry extract compared to control samples throughout the 16–40 days of refrigerated storage was reported. The growth of other bacteria tested was also inhibited, whilst the shelf-life of pork hamburgers was extended from 5 to 9 days. Furthermore, cranberry pomace extract showed antioxidant activity in hamburgers during storage. Regarding the impact of the extract on organoleptic properties; a significant (*p* < 0.05) effect on colour intensity in pork burgers and cooked ham was observed, whereas no significant (*p* > 0.05) impact on discolouration, odour and flavour intensity and juiciness of cooked ham was reported. In overall, the sensory properties of pork burger and cooked ham were not negatively affected.

Similar results have been observed in another study by Wu et al. [138]. Cranberry concentrate (2.5–7.5% *w*/*w*) was incorporated into ground beef meat and a reduction of total aerobic bacteria and *E. coli* O157:H7 after 5 days cold storage was observed. In that study the organoleptic properties of the burgers were evaluated and no effect of the cranberry concentrate was observed, at concentrations up to 5% *w*/*w*. The burgers with the 7.5% *w*/*w* cranberry concentrate exhibited a slightly sour taste.

### 2.3. Organic Acids

The majority of organic acids are natural, being part of plant or animal metabolism; they can be found naturally in food products and they have the ability to inhibit the growth of microorganisms. Hence, they are traditionally used as preservatives of food products. Several organic acids, their salts and anhydrides are generally recognized as safe (GRAS) by the FDA and are listed as food preservatives in European legislation [12,168,169]. Apart from their anti-microbial activity, organic acids can play a key role in food products as stabilizers, antioxidants and flavouring enhancers [170].

The anti-microbial activity of organic acids, such as citric acid and acetic acid, is based on reducing the pH of the food, leading to inhibition of microbial growth [170]. Moreover, the undissociated form of organic acids can penetrate the cell membrane and dissociate inside the cell because of the high pH. Then, the pH inside the cell will be decreased, leading to disruption of enzymatic and nutrient transport systems. The protons that will be produced by the organic acids due to the dissociation, should be transported outside of the cell in order to keep the pH inside them neutral and this transportation process is energy-consuming for the cells, exhausting their energy sources [171].

Many organic acids have been added to food products or to packaging films and coatings with the aim of prolonging shelf-life and controlling the growth of microorganisms.

Lactic acid has been studied thoroughly as an anti-microbial agent. Smulders et al. [172] studied if the combination of polyamide-based film with lactic acid and vacuum packaging could extend the shelf-life of beef. The results showed that the population of *E. coli* O157:H7 was 2 log units lower compared to untreated samples after storage at 12 °C for 14 days. Jaspal et al. [44] studied the combined effect of treatment with a lactic acid solution and packaging on quality of chicken. The application of 1.25% lactic acid in combination with MAP led to a significantly (*p* < 0.05) lower population of total aerobic counts during refrigerated storage. In addition, preservation of meat colour and reduction of lipid oxidation during storage of the samples treated with lactic acid and modified atmosphere packaging was also reported. Regarding the organoleptic properties, odour and taste of cooked chicken with lactic acid were considered acceptable by the panellists who participated in the study.

De Oliveira et al. [173] dipped meat in lactic and acetic acid solutions, at concentrations equal to their MIC (2.5 µL/mL and 0.6 µL/mL, respectively), with the purpose of testing the anti-microbial efficacy of these solutions against *S. aureus*. Both organic acids were effective, however acetic acid demonstrated slightly better anti-microbial activity in comparison with lactic acid throughout the incubation period at 7 °C. Moreover, application of the same solutions to meat broth showed better results compared to meat.

The salts of lactic acid are also considered effective in extending the shelf-life of food products. Sodium lactate was added to thermally processed meats as a preservative and it reduced the population of lactic acid bacteria by approximately 3 log CFU/g compared to control samples after 40 days storage at 4 °C. However, the addition of high levels of salt (3%) led to a strong salty taste [174]. In another study, sodium lactate application on pork sausages led to an extension of shelf-life of approximately 4 days compared to the control, whilst no significant (*p* > 0.05) differences in the organoleptic properties of the sausages were observed [175].

Citric acid has been incorporated into packaging materials as an anti-microbial agent. Battisti et al. [176] incorporated citric acid into gelatin-coated paper in order to pack beef. The total microbial population in the samples packed with the active packaging was significantly (*p* < 0.05) lower by approximately 3 log CFU/g compared to control samples after 4 days of storage at 4 °C. The different concentrations (0.5 and 1%) of citric acid used did not influence the anti-microbial efficacy of the active paper. In another study, Júnior et al. [177] incorporated citric acid into a biodegradable packaging composed of cornstarch and linear low-density polyethylene (LLDPE). This active packaging was used for packing beef. Reduction of total bacterial count by 1 log CFU/g compared to control samples after 10 days of cold storage at 4 °C was reported.

An organic acid that has been extensively used as a preservative is sorbic acid. Limjaroen et al. [178] incorporated sorbic acid, at level of 1.5% and 3%, into polyvinylidene chloride (PVDC) films intended for packaging of sliced bologna. The anti-microbial film reduced the growth of *L. monocytogenes*, mesophilic aerobic bacteria and lactic acid bacteria on bologna after the 28 days chilled storage. The incorporation of sorbic acid (1.5%) into Argentine anchovy protein film and the use of this film for packaging beef led to inhibition of *E. coli* O157:H7 and *L. monocytogenes*. Specifically, a reduction of 5 log and 4 log CFU/g in *E. coli* O157:H7 and *L. monocytogenes*, respectively, compared to the control film was observed after 12 days of storage at 5 °C [179]. Benzoic acid, another natural organic acid, was also incorporated from da-Rocha et al. [163] into Argentine anchovy protein film in this study. The result was a decrease of 6 log and 5 log CFU/g in *E. coli* O157:H7 and *L. monocytogenes*, respectively, in beef packaged with the anti-microbial film compared to the control film, during the same storage conditions (12 days at 5 °C).

Caprylic acid (octanoic acid), is an organic acid that belongs to the fatty acids. The anti-microbial mechanism of the fatty acids is not fully understood; however, they target the bacterial membrane and it is thought they may affect the electron transport chain and oxidative phosphorylation, inhibit enzymes, disrupt nutrient uptake, produce products that possess anti-microbial properties by auto-oxidation or peroxidation secondary degradation, increase the permeability of the membrane and provoke the lysis of cells [180]. Caprylic acid has been used in studies as an anti-microbial agent. Moschonas et al. [43] added it, at concentrations of 0.25–1%, in not-ready-to-eat surface-browned, breaded chicken which was stored at −20 °C. The result was a significant (*p* < 0.05) reduction of the population of *Salmonella* sp., by 1 to 4.5 log CFU/g, in the chicken after 7 days frozen storage.

Sodium octanoate (SO), which is a derivative of caprylic acid, demonstrates also anti-microbial properties. Reid et al. [181] incorporated it, at concentrations 2.5 and 3.5 times the MIC, into gelatin coated film and beef was packaged in it. Growth inhibition of *Clostridium estertheticum*, leading to significant (*p* < 0.01) delay of blown pack spoilage of beef was reported. At the same study, Auranta FV (AFV), a commercially used anti-microbial, was also incorporated into the active packaging. This anti-microbial is a mixture of organic acids (citric, malic, lactic and caprylic acids) with bioflavonoids. The results were very promising since the onset of the blown pack spoilage of beef was delayed from approximately 28 days in the control samples to 48 days for the treated packs. Clarke et al. [182] coated sodium octanoate and Auranta FV onto the inner polyethylene layer of cold plasma treated polyamide/low-density polyethylene (PA/LDPE) films. The anti-microbial packaging was used for packing beef steaks. The shelf-life of beef steaks packed with the AFV and SO coated films was significantly (*p* < 0.05) extended by 33% or 55%, respectively, compared to samples packed with the control films.

Inbac^TM^ is another commercially available anti-microbial agent, which is based on organic acids and it has been used in recent research studies. It is composed of sodium acetate (43%), malic acid (7%), emulsifier-mono and diglycerides of fatty acids and technological coadjuvants; anticaking agents, calcium phosphate, magnesium carbonate and silicon dioxide (~50%). O’ Neill et al. [183] studied the impact of Inbac^TM^ (0.3%) combined with high pressure processing (HPP) (580 MPa) on shelf-life of vacuum-packed salt reduced frankfurters and cooked ham stored at 4 °C. The application of HPP and Inbac^TM^ led to prolongation of shelf-life of frankfurters and cooked ham by 51% and 97%, respectively, compared to control samples which contained full salt content. When the Inbac^TM^ was used alone, the low-salt processed meat products reached the limit of acceptability faster in comparison with the control samples.

### 2.4. Animal-Derived Anti-Microbial Compounds

#### Chitosan

Chitosan is a polysaccharide biopolymer that is produced by partial alkaline N deacetylation of chitin, which is obtained from shrimp and crab shells and its characteristics, such as biodegradability, biocompatibility and nontoxicity make it ideal for several applications [184]. It also has anti-microbial properties [185], so, it can be used for packaging of food products alone or in combination with other anti-microbial compounds in order to extend their shelf-life.

The anti-microbial mode of action of chitosan against *S. aureus*, and against Gram-positive bacteria in general, as was studied by Raafat et al. [184] is based on its ability to bind to the cell wall. It disrupts the bacterial membrane, leading to leakage of cellular content and deformation of the cell. Furthermore, the pathways in the cell membrane that are responsible for energy production are also disrupted and as a result the cells start to produce energy anaerobically. The anti-microbial mechanism and the effect of chitosan in *S. aureus* at molecular level was also investigated. Chitosan induced the downregulation of genes involved in RNA and protein synthesis, metabolism of carbohydrates, amino acids, nucleotides and nucleic acids, lipids and coenzymes.

Chitosan is a well-known food coating and many researchers have studied its effectiveness on various food products. In one study beef was packed with chitosan membrane combined with nanofibers. This anti-microbial film showed an 85% reduction of the *L. monocytogenes* population after 24 h at 4 °C [52]. Sogut and Seydim [141] used chitosan in order to pack chicken meat and they observed that the mesophilic bacteria and coliform bacteria were reduced by approximately 1.5 log and 2.6 log CFU/g in the treated samples compared to the control, after 15 days of refrigerated storage.

### 2.5. Bacteriocins

Bacteriocins are produced by lactic acid bacteria and they are a group of proteinaceous bioactive bacterial substances, which demonstrate anti-microbial activity against other bacteria [186]. Successful applications of bacteriocins in active packaging of meat products are shown in Table 4.

Nisin is a typical example of a bacteriocin. It is an anti-microbial peptide produced by *Lactococcus lactis* subsp. *lactis* and it is active against Gram-positive bacteria, as well as being able to inhibit the outgrowth of spores of *Bacillus* and *Clostridium* spp. Nisin is a GRAS substance, approved as an additive by FAO and it has a significant role in the food industry as a natural preservative for different types of food products [190,194,195]. The anti-microbial mechanism of nisin targets the cell membrane. Nisin can bind to phospholipids, such as the pyrophosphate moiety of lipid II, of the bacterial cell membrane and then it creates pores and inhibits cell wall biosynthesis. The creation of pores reduces the proton motive force and has a detrimental effect on the membrane electrical potential and pH gradient, leading eventually to cell death [196,197].

Nisin has been extensively studied as an anti-microbial agent in active packaging. La Storia et al. [187] used a nisin based anti-microbial solution, which was coated on HDPE film. This anti-microbial film, combined with MAP (60% O_2_:40% CO_2_), was used for packaging of beef steaks. They observed that the combination of anti-microbial packaging with MAP led to significantly lower levels of spoilage microorganisms in beef steaks (*p* < 0.05) after 12 days in cold storage. At the same time, the sensory properties of the meat were not affected by the packaging. Nisin has also been incorporated in collagen casings for sausages. Batpho et al. [190] observed that this anti-microbial casing had an anti-listerial effect, causing 90% reduction of *L. monocytogenes* in sausages throughout the storage period at 4 °C and extended the shelf-life to at least 90 days at 4 °C.

Pediocin belongs to the Class II of bacteriocins, it is a small anti-microbial peptide that is produced by *Pediococcus* genera and demonstrates bactericidal effect against Gram-positive bacteria [198]. The mechanism of pediocin against *Pediococcus* has been studied by Chikindas et al. [199]. Pediocin targets the cell membrane, creates pores in the membrane, causes dissipation of the electrical potential, inhibits the transport of amino acids and block the uptake of nutrients and cause leakage of ions, amino acids and other molecules from the cells.

Pediocin has been used as an anti-microbial agent aimed at extending the shelf-life of meat products. Santiago-Silva et al. [192] incorporated pediocin, at different concentrations, into cellulose-based film and they studied the efficacy of this active packaging on sliced ham. A 2 log CFU/g reduction in the population of *L. innocua* compared to control after 15 days of storage at 12 °C was observed when a high concentration (50% *w*/*w*) of pediocin was used. On the other hand, the decrease of *Salmonella* sp. was negligible throughout the storage period irrespective of the concentration of pediocin that was used.

Sakacin-A, is produced by *Lactobacillus sakei* and it is a peptide with anti-listerial activity. Its anti-microbial mechanism against *L. monocytogenes* is focused on the cell membrane, and it has dual activity [200]. Firstly, it can dissipate the proton motive force and the pH gradient, leading to leakage of cellular materials and secondly it disrupts the cell walls by breaking specific bonds in the peptoglycan structure.

Sakacin-A has been used in active packaging studies. Barbiroli et al. [193] coated PE-coated paper sheets with a Sakacin-A based solution. The bacteriocin-activated film was used for packaging thin slices of beef carpaccio. The results showed that the population of *L. innocua* was approximately 1.5 log CFU/g lower in comparison to the control sample after 48 h at 4 °C.

## 3. Synergism Between Anti-Microbial Compounds

The combination of anti-microbial agents could lead to synergistic, additive or antagonistic effects. A synergistic effect means that the combination of anti-microbial agents has greater efficacy in comparison with the sum of the individual agents alone. An additive effect is observed when the anti-microbial efficacy of the combined agents is equal to the sum of the individual effects. On the other hand, antagonism occurs when the combined agents are less effective compared to the effect they would have if they had been applied separately [27]. It is not known what exactly regulates the above effects in food products. It can be assumed that a synergistic effect can be produced when two anti-microbials agents with different anti-microbial mode of actions are combined [201].

In this section of the review, a summary of the studies, where a synergistic effect between anti-microbial agents occurred, is presented.

Raeisi et al. [54] studied the anti-microbial efficacy of sodium alginate coating containing different combinations of rosemary EO, cinnamon EO and nisin against several microorganisms on chicken meat at 4 °C. All combinations proved effective in inhibiting the growth of the microorganisms tested. However, the combination of rosemary EO (5 mg/mL) with cinnamon EO (5 mg/mL) demonstrated the higher efficacy and prolonged the shelf-life of chicken for 6 days compared to the control.

Nisin has also been used in combination with other anti-microbial compounds for increasing the shelf life of food products and inhibiting the growth of pathogenic bacteria. Theivendran et al. [142] observed that the combination of nisin (10,000 IU) with either green tea extract (1%) or grape seed extract (1%), incorporated in soy protein edible film, was very effective against *L. monocytogenes* on turkey frankfurters. A 2.7 log CFU/mL decrease of the pathogen compared to the control was observed after 28 days at 4 °C, when nisin was combined with either GTE or GSE. A similar reduction compared to the control (2.3–2.5 log CFU/mL) was also observed after storage of the turkey frankfurters for 28 days at 10 °C. When the soy protein film containing 1% GSE or 1% GTE was coated on the turkey frankfurters, no anti-microbial effect was observed, whereas the film with nisin (10,000 IU) alone was only effective against *L. monocytogenes* at low temperature.

Olaimat and Holley [202] incorporated allyl-isothiocyanate (25 µL/g or 50 µL/g) or mustard extract (100 mg/g or 250 mg/g) into acidified (1.5% malic or acetic acid) k-carrageenan/chitosan-based coatings. These coating were used on cooked, cured roast chicken slices stored under vacuum at 4 °C. All of the combinations led to significantly (*p* < 0.05) lower populations of *L. monocytogenes*, aerobic bacteria and lactic acid bacteria on cooked, cured chicken throughout the 70 days of cold storage. The coating with 1.5% (*w*/*v*) malic acid were more effective compared to the ones acidified with acetic acid, whereas the coatings with allyl-isothiocyanate demonstrated higher efficacy compared to the coatings containing mustard extract.

Talebi et al. [59] incorporated essential oil from black zira and peppermint at different levels into polylactic acid-nanocellulose film. The combination of 1% *v*/*v* black zira EO with 1% *v*/*v* peppermint EO had a higher anti-microbial efficacy compared to all the different combinations of concentrations of EO tested, leading to an extension of shelf-life from 4 to 7 days. On the other hand, a negative impact on the colour of the meat was observed.

Gammariello et al. [203] studied the synergistic effect of essential oils of fennel, black pepper, bay and nutmeg, sodium lactate and packaging under modified atmosphere conditions on the extension of the shelf-life of sausages. The results showed that the treatments were effective in limiting the growth of bacteria. Their shelf-life at 4 °C was extended from 2 to 18 days (increase of 859%), when the combination of bay EO (2.5%), nutmeg EO (1.25%), sodium lactate (60%) and packaging under MAP (30% CO_2_:70% N_2_) was applied to the sausage. In addition, an extension of shelf life of the sausage from 2 to 14 days (increase of 620%) was observed when 1.25% fennel and 2.5% black pepper essential oils, sodium lactate (60%) and packaging under MAP (30% CO_2_:70% N_2_) were combined.

Wang et al. [204] incorporated mixtures of cinnamon EO with ginger EO (ratio 1:1), at concentrations 0.05%, 0.2% and 1%, into chitosan-based films in order to package pork. The application of the film at all concentrations tested reduced the growth of total aerobic bacteria throughout the storage period of 9 days at 4 °C. The inhibition of bacterial growth was higher when a higher concentration of the mixture of essential oils was used. In addition, the application of this anti-microbial film slowed down lipid oxidation in the meat.

Derivatives of cinnamon, such as cinnamate or cinnamic acid can be used in combination with other anti-microbial agents in order to increase the shelf-life of meat products. According to Saurabh et al. [205], cinnamate components, at concentration 1–50% *w*/*w*, combined with vanillin components, at concentration 0.5–25% *w*/*w*, with weight ratio between them in the range of 0.1–0.5, can inhibit the growth of bacteria and extend the shelf-life of meat products.

Hop extracts can be used in combination with other anti-microbial agents and have a synergistic effect in inhibition of bacteria in meat products. Berdahl et al. [206] used hop extract containing β-acids, combined with an extract of any herb of *Lamiaeae* family, preferably rosemary that contains carnosic acid, carnosol, rosmarinic acid or any mixtures of them in order to extend the shelf-life of fresh meat. The presence of oxygen, at least 20%, was mandatory for the synergistic effect of the herbs extracts and they identified that the weight ratio of acids from hop and from any herb of *Lamiaceae* should always be between 0.5 and 13. This synergistic effect could also preserve the desired colour of the meat, whilst rosemary extract (0.1%) could effectively cover the bitter flavour of hop extract in levels up to 100 ppm when added to ground beef with 30% fat content.

## 4. Nanotechnology and Nanomaterials

The potential for use of Nanotechnology and anti-microbial nanoparticles is gaining increased attention in the food industry.

Nanotechnology involves the manipulation of matter at extremely small scale, which is generally between 1 and 100 nm. Nanomaterials and nanoparticles may be observed in the forms of nanoparticles, nanotubes, fullerenes, nanofibers, nanowhiskers and nanosheets [207]. The EU defines, “nanomaterial” as “a natural, incidental or manufactured material containing particles, in an unbound state or as an aggregate or as an agglomerate and where, for 50% or more of the particles in the number size distribution, one or more external dimensions is in the size range 1 nm–100 nm” [208].

Nanotechnology can be used to protect and deliver anti-microbial agents in food products. Nano-encapsulation of bioactive compounds such as essential oils and plant extracts has several advantages, such as increased bioavailability, high loading capacity, high solubility, improved stability, controlled release of bioactive compounds and the ability to mask potential unwanted flavours [209]. Successful applications of nano-encapsulation of anti-microbial agents have been reported in several studies and they include nanogels [56], nanofibers [52], nanofibers with cyclodextrin inclusion [53], nano-sized solubilisates [210], nano-emulsions [40] and carbon nanotubes [63] among others.

Metal ions and nanoparticles (NPs) are natural anti-microbial agents that exhibit strong anti-microbial activity [211,212,213]. The anti-microbial mode of action of nanoparticles can be attributed to several different mechanisms, such as inhibition of cell wall and/or membrane synthesis, disruption of energy transduction, production of toxic reactive oxygen species that provoke oxidative stress, photocatalysis, inhibition of enzymes and lower DNA production [214]. Their application in food packaging materials has been also tested. Zinc oxide (ZnO) nanoparticles were incorporated into calcium alginate film prepared for packaging poultry meat sausages. The populations of *S. aureus* and *S.* Typhimurium could not be detected after 6 and 8 days of storage at 8 °C, respectively [213]. Azlin-Hasim et al. [212,215] incorporated silver (Ag) into polyvinyl chloride (PVC) and low-density polyethylene (LDPE) nanocomposite films and used the films for packaging chicken breast fillets under MAP (60% N_2_:40% CO_2_). In those studies, a significant (*p* < 0.05) extension of shelf-life of the chicken fillets packaged in the anti-microbial film was reported, whilst the lipid oxidation was also reduced. Moreover, it was observed that Gram-negative bacteria were more susceptible to Ag nanoparticles compared to Gram-positive bacteria. Hence metal nanoparticles could potentially be an alternative and effective approach for enhancing the safety of meat products.

Regarding the regulations of using nanomaterials and nanoparticles, it should be mentioned that some salts of silver have been approved by the FDA for use in packaging materials of food products [216]. On the other hand, according to the regulation EC 450/2009, the use of nanoparticles in Europe is assessed on a case-by-case basis with regards to their risk until more information is known about this technology, [19]. To assess the risk, data for the overall and specific migration from the packaging materials containing the nanoparticles to the food product is needed [217]. According to European Food Safety Authority (EFSA), the overall migration of silver nanoparticles should not exceed 0.05 mg/kg in food products [218]. Hence, additional research regarding the exposure assessment and toxicity of the nanomaterials should be carried out before their application [219].

## 5. Regulations, Limitations and Future Perspectives

The regulations about food additives are generally strict. Most of the natural anti-microbial compounds originating from plant extracts, essential oils organic acids, etc. are included in the list of food flavouring substances of the European Union, according to regulation EC 872/2012 and some of them have also been approved as food additives in meat products, according to regulation EC 1333/2008 [12,34]. Furthermore, according to regulation EC 450/2009 [19], the anti-microbial agents used in active packaging which will be released into food must comply with regulation EC 1333/2008, which means they have to be included in the list of approved food additives. In the USA, the majority of the natural anti-microbial agents are considered as Generally Recognized as Safe (GRAS) according to the FDA [33].

Any natural anti-microbial compounds that will be commercially used in meat products should be licensed for use in humans. Safety risk assessments regarding potential adverse effects on human health need to be conducted. In the case of European Union, EFSA will issue an opinion on the safety of the proposed substances and its intended use. All of this process, which starts by applying and providing well documented scientific evidence about the safety and the benefits of this specific substance that will be used is time consuming and costly [27,220,221]. Hence, this is a limitation that has to be addressed by the meat industry.

Another limitation, regarding the regulations is that they differ across the world. An example is estragole, which is not permitted as an additive in food products in Europe, because it is carcinogenic and genotoxic [222]. On the other hand, the FDA in the USA has approved its use [33].

The stability of plant extracts, especially of essentials oils, at high temperatures and their poor solubility in water are some limitations for their use in meat products. For example, cinnamaldehyde, the main compound of cinnamon EO, is broken down to benzaldehyde, during heating at 60 °C, whilst it is stable for 30 min at 200 °C during heating in combination with eugenol or cinnamon leaf oil [223]. Encapsulation could solve this problem. The anti-microbial agent will be entrapped, thus its solubility and stability during processing will be enhanced [224]. Nanotechnology could also enhance the bioavailability of anti-microbial agents in meat products, by nano-encapsulation; however, as was mentioned above additional research regarding the exposure assessment and toxicity of the nanomaterials should be carried out [219].

The synergistic effect between different anti-microbial agents, but also potential synergistic effect with other preservation methods, such as high-pressure processing (HPP), should be further studied. This synergism will help to reduce the amount of anti-microbial agents needed, leading to minimum impact on organoleptic properties, maximum efficacy and cost reduction. It should be also mentioned that the cost of using essential oils is quite high [31]. A natural preservation method for meat products has to be not only technically feasible but also cost-effective.

Finally, it has been observed that consumers demand food products with high health and nutritional value. Meat and meat products are a category of products with positive and negative nutritional properties [225]. Nevertheless, they could be fortified with functional ingredients that promote health and improve their nutritional profile. Several of the anti-microbial agents that were presented in this review are considered as health promoting substances. A typical example is the plant extracts that are rich in polyphenols and flavonoids. Flavonoids have anti-oxidative, anti-inflammatory, anti-mutagenic and anti-carcinogenic properties, whilst they can regulate cellular enzyme functions [226], hence the addition of them to the product or in the packaging material will lead to a functional meat product with enhanced health-related properties.

## 6. Conclusions

Since consumers demand “clean label” food products that are free of synthetic preservatives, food industries and scientists need to identify alternative, green preservation methods. As presented in this review, natural anti-microbial agents could provide and effective alternative for ensuring the safety and increasing the shelf-life of meat and processed meat products.

Despite the numerous studies that prove their efficacy, natural anti-microbials are not yet widely used. Limitations with the legislation, regarding the use of natural anti-microbials in meat products and in the packaging material need to be overcome. Moreover, further investigation of nanomaterials and its impact on human health, potential interactions of anti-microbial compounds with the meat components that affect their efficacy and potential synergistic activity between natural anti-microbial agents and other preservation methods should be conducted. Nevertheless, using natural anti-microbials not only will enhance the quality and safety of meat products in the future but it will also be useful for marketing purposes and increasing sales. Naturally derived anti-microbials will meet the expectations of consumers for “clean label”, nutritious and safe meat products.

## Figures and Tables

**Table 1:** Overview of studies testing the efficacy of essential oils and plant extracts or their components when added to meat products.

Anti-Microbial Agent—Concentration Applied	Food Product	Microorganisms Targeted	Indicative Reduction (Log_10_ CFU/g, Log_10_ CFU/mL, Log_10_ CFU/cm^2^) ^1,2,3,4^	Reference
Bay—0.5% *v*/*w*	Ground chicken	Total viable counts	0.14–0.47	[35]
		*Listeria monocytogenes*	0.07–0.36	
		*Escherichia coli*	0.12–1.22	
Cinnamon—1% *w*/*w*	Marinade on chicken	Lactic acid bacteria	0.5	[36]
		Yeasts and moulds	0.8	
		*E. coli*	0	
		Coliforms	0	
1% *w*/*w*	Marinade on pork	Lactic acid bacteria	0	[36]
		Yeasts and moulds	2.4	
		*E. coli*	0	
		Coliforms	0	
Clove—0.5%, 1%	Minced beef	*Aspergillus flavus*	2.42–3.01 ^5^	[37]
Coriander—0.02% *v*/*w*	Ground beef	Total viable counts	0.1–0.4	[38]
		*Enterobacteriaceae*	1–1.5	
		Lactic acid bacteria,	0–0.8	
		Total anaerobic counts	0.5	
Fennel—0.2% *v*/*w*	Chicken	Total viable counts	0.9	[39]
		*Enterobacteriaceae*	2.4	
		Lactic acid bacteria	0	
Garlic—0.5%, 1%	Minced beef	*A. flavus*	1.93–2.12 ^5^	[37]
Ginger—3%, 6% *w*/*w*	Chicken	Psychrophilic bacteria	2.7–4.5 ^6^	[40]
		Yeasts and moulds	0.9–3.3 ^6^	
Hop—5000 ppm	Model marinade on pork	Mesophilic bacteria	0.9	[41]
		*Enterobacteriaceae*	0	
		*Listeria monocytogenes*	1.5	
		Lactic acid bacteria	1.3	
Hyssop—0.02% *v*/*w*	Ground beef	Total viable counts	0–0.3	[38]
		*Enterobacteriaceae*	0.9–1	
		Lactic acid bacteria	0.1	
		Total anaerobic counts	0.5	
Nutmeg—10, 20 ppm	Cooked Sausage	Total mesophilic bacteria	0.13–0.38 ^7^	[42]
Oregano—0.3%, 0.4%, 0.5% *v*/*w*	Chicken	*Salmonella* sp.	2.7–3.8	[43]
0.2% *w*/*w*	Chicken	Lactic acid bacteria	0.67–0.86	[44]
Pepper—0.1%, 0.5% *v*/*v*	Pork	*Pseudomonas* spp.	2.1–3.05	[45]
		*Enterobacteriaceae*	1.05–1.85	
		*Brochothrix* spp.	0	
		Lactic acid bacteria	0	
Peppermint—0.25%, 0.5% *v*/*w*	Minced beef	Total aerobic bacteria	1.44–1.58	[46]
		Psychrotrophic bacteria	1.36–1.4	
		*Enterobacteriaceae*	0.3–1.2	
		*Pseudomonas* spp.	1–1.11	
Rosemary—2% *v*/*w*	Chicken	*Pseudomonas aeruginosa*	0	[47]
		Lactic acid bacteria	2.57	
0.2% *v*/*w*	Chicken	Anaerobic bacteria	1.05	[48]
		*Enterobacteriaceae*	3.62	
		Lactic acid bacteria	0.69	
		*Pseudomonas* spp.	2.72	
Sage—0.2% *v*/*w*	Chicken	Anaerobic bacteria	1.13	[48]
		*Enterobacteriaceae*	3.62	
		Lactic acid bacteria	0.81	
		*Pseudomonas* spp.	2.72	
0.1% *w*/*w*	Mechanical Separated Meat (MSM) from chicken	Mesophilic bacteria	1.2	[49]
		Psychrotrophic bacteria	0.3	
		*Enterobacteriaceae*	2.4	
		Coliforms	2.3	
		Enterococci bacteria	1.4	
Savory—0.2% *v*/*w*	Chicken	Total viable counts	0.8	[39]
		*Enterobacteriaceae*	1	
		Lactic acid bacteria	0	
Sweet lemon—500 ppm	Sausage	Total viable count	0.03	[50]
		*Pseudomonas* spp.	0.05	
		Psychrotrophic bacteria	0.04	
		Lactic acid bacteria	0.08	
		*Enterobacteriaceae*	0.11	
Thyme—500 ppm	Sausage	Total viable count	1.75	[50]
		*Pseudomonas* spp.	0.85	
		Psychrotrophic bacteria	1.69	
		Lactic acid bacteria	1.18	
		*Enterobacteriaceae*	0.09	
0.5%, 1%	Minced beef	*A. flavus*	2.25–2.64 ^5^	[37]

The synergistic effect of anti-microbial agents or their component, where it has been studied, is not cited. ^1^ The difference of population that is cited is sometimes an approximate number, because of lack of exact data; ^2^ Where the range of population difference is cited it corresponds to the different experimental conditions studied (e.g., different concentrations, storage temperatures, etc.); ^3^ Where the anti-microbial agents were combined with other preservation methods, the effect of the agent alone is cited; ^4^ These classifications apply to the end point of each experiment; ^5^ The population difference cited corresponds to the 3rd day of the experiment, because of lack of data for the control sample; ^6^ The population difference cited corresponds to the 8th day of the experiment, because of lack of data for the control sample; ^7^ The population of total mesophilic bacteria was converted from CFU/g to Log_10_ CFU/g.

**Table 2:** Overview of studies testing the efficacy of anti-microbial packaging with essential oils and plant extracts or their components on meat products.

Anti-Microbial Agent—Concentration Applied	Food Product	Packaging Material	Microorganisms Targeted	Indicative Reduction (Log_10_ CFU/g, Log_10_ CFU/mL, Log_10_ CFU/cm^2^) ^1,2,3,4^	Reference
Angelica Root—0.1% *v*/*w*	Pork	Polyethylene (PE) film	Total counts	2.34	[51]
			*Brochothrix thermosphacta*	1.49	
			*Carnobacterium* spp.	1.04	
			*Enterobacteriaceae*	1.95	
			*Staphylococcus* sp.	1.17	
			*Pseudomonas* sp.	0.89	
			*Enterococcus* sp.	0.81	
Chrysanthemum—1.5% *w*/*v*	Beef	Chitosan/nanofibers film	*L. monocytogenes*	1.2–1.5	[52]
Cinnamon—10% *w*/*w*	Pork	Polylactic acid (PLA)/b-cyclodextrin nanofilm	Total viable counts	4.5 ^5^	[53]
5 mg/mL	Chicken	Sodium alginate (NaAlg) coating	Total viable counts	1.4	[54]
			Psychrotrophic bacteria	1	
			*Pseudomonas* spp.	0.8	
			*Enterobacteriaceae*	0.6	
			Lactic acid bacteria	1.5	
			Yeasts and moulds	0.5	
			*L. monocytogenes*	1	
Clove—0.1% *v*/*w*	Pork	Polyethylene (PE) film	Total counts	2.49	[51]
			*B. thermosphacta*	2.17	
			*Carnobacterium* spp.	1.62	
			*Enterobacteriaceae*	2.37	
			*Staphylococcus* sp.	1.73	
			*Pseudomonas* sp.	1.34	
			*Enterococcus* sp.	1.26	
3%	Ground chicken meat	Carboxymethyl cellulose–polyvinyl alcohol (CMC-PVOH) film	Total viable counts	3 ^6^	[55]
1, 2 mg/g	Beef	Chitosan-myristic acid nanogel coating	*Salmonella enterica* ser Enteritidis	0.3–0.8	[56]
0.5 g in 9 × 5 cm film	Ground chicken	Linear-low density polyethylene (LLDPE) film	*S. Typhimurium*	7	[57]
			*L. monocytogenes*	6	
Coriander—0.5%, 1%	Chicken	Sodium alginate (NaAlg)-glycerol coating	Mesophilic bacteria	1–1.5	[58]
			Psychrotrophic bacteria	0.8–1.2	
			Lactic acid bacteria	1.3–2.3	
			Coliforms	1.4–2.4	
			*Staphylococcus aureus*	1.2–2.1	
			Moulds and yeasts	1.1–1.5	
Cumin—Black zira—0.5%, 1% *v*/*v*	Ground beef	Polylactic acid (PLA)-nanocellulose (NC) film	Total viable bacteria	0.7	[59]
			Lactic acid bacteria	0.1–0.2	
			*Enterobacteriaceae*	0.5–0.8	
			Psychrotrophic bacteria	0.2–0.3	
			*S. aureus*	0.6–1.2	
			*Pseudomonas* spp.	0.1–0.3	
0.5%, 1%, 2% *v*/*v*	Chicken	Chitosan coating	Mesophilic bacteria	0.45–0.59	[60]
			Psychrotrophic bacteria	0	
			Lactic acid bacteria	0	
			*Enterobacteriaceae*	0	
			Yeasts and moulds	0	
Eucalyptus—0.5%, 1%, 2% *v*/*v*	Chicken	Chitosan coating	Mesophilic bacteria	0–0.69	[60]
			Psychrotrophic bacteria	1.11–1.41	
			Lactic acid bacteria	0	
			*Enterobacteriaceae*	0	
			Yeasts and moulds	0	
Garlic—2%, 4%, 6%, 8% *w*/*w*	Ready-to-Eat (RTE) beef loaf	Low density polyethylene (LDPE) film	*L. monocytogenes*	0.2–0.5	[61]
			*E. coli*	0–0.2	
			*B. thermosphacta*	0–0.2	
Horseradish, mustard—0.6, 1.2 µg/h release rate	Chicken	Glass vial inside high density polyethylene (HDPE) film	*S. enterica* ser Typhimurium	1–1.2	[62]
			*L. monocytogenes*	0.1–0.6	
			Total aerobic bacteria	0.5–1	
20%, 28%, 40%, 52%, 58% *v*/*w*	Cooked chicken	Cellulose film with carbon nanotube (CNT)	*Salmonella* Choleraesuis	2.5–8	[63]
			Psychrotrophic bacteria	1.5–8	
			Mesophilic bacteria	0.7–8	
Lemongrass—2%	Pork sausage	Polylactic acid (PLA) film	*L. monocytogenes*	1.47	[64]
Nutmeg—0.5%, 1%, 1.5%, 2%	Beef	Sage seed mucilage coating	Total viable count	3–8	[65]
			Psychrotrophic bacteria	1.8–5.5	
			*E. coli*	0.5–2	
			*S. aureus*	1–3	
			Yeasts and moulds	1–5	
Oregano—1%, 2%, 3% *v*/*v*	Beef	Soy protein coating	*E. coli* O157:H7	1.2–1.83	[66]
			*L. monocytogenes*	0.9–1.9	
			*S. aureus*	1.8–2.86	
5% *v*/*v*	Ground beef patties	Soy protein film	Total viable count	0	[67]
			Lactic acid bacteria	0	
			*Staphylococcus* spp.	0	
			*Pseudomonas* spp.	0.6	
			Coliforms	0.5	
Pepper—2% *w*/*w*	Chicken	Carboxymethyl cellulose (CMC) coating	Mesophilic bacteria	1.5–2.2	[68]
			Psychrotrophic bacteria	1.5–2	
Peppermint—0.5%, 1% *v*/*v*	Ground beef	Polylactic acid (PLA)- nanocellulose (NC) film	Total viable bacteria	0.7	[59]
			Lactic acid bacteria	0–0.4	
			*Enterobacteriaceae*	0.6–0.8	
			Psychrotrophic bacteria	0.4	
			*S. aureus*	0.6–0.8	
			*Pseudomonas* spp.	0.1–0.4	
Rosemary—4% *w*/*w*	Chicken	3 layers film (paper-metallic layer- high density polyethylene (HDPE))	Mesophilic bacteria	0.3	[69]
			*Enterobacteriaceae*	0	
			*Pseudomonas* spp.	0.24	
			*B. thermosphacta*	0.37	
5 mg/mL	Chicken	Sodium alginate (NaAlg) coating	Total viable counts	1.6	[54]
			Psychrotrophic bacteria	1.6	
			*Pseudomonas* spp.	1.3	
			*Enterobacteriaceae*	1.6	
			Lactic acid bacteria	1.7	
			Yeasts and moulds	0.8	
			*L. monocytogenes*	1.5	
0.1% *v*/*w*	Pork	Polyethylene (PE) film	Total counts	2.53	[51]
			*B. thermosphacta*	1.95	
			*Carnobacterium* spp.	1.5	
			*Enterobacteriaceae*	2.48	
			*Staphylococcus* sp.	1.39	
			*Pseudomonas* sp.	1.08	
			*Enterococcus* sp.	0.98	
4% *w*/*w*	Beef	3 layers film (paper-metallic layer- high density polyethylene (HDPE))	Psychrotrophic bacteria	0–0.7 ^7^	[70]
			*B. thermosphacta*	0–1.5 ^7^	
			*Pseudomonas* spp.	0–0.8 ^7^	
			*Enterobacteriaceae*	0–0.8 ^7^	
Thyme—1%, 2%, 3% *v*/*v*	Beef	Soy protein coating	*E. coli* O157:H7	1.2–1.87	[66]
			*L. monocytogenes*	1–1.97	
			*S. aureus*	1.6–2.6	
5% *v*/*v*	Ground beef patties	Soy protein film	Total viable count	0	[67]
			Lactic acid bacteria	0	
			*Staphylococcus* spp.	0.2	
			*Pseudomonas* spp.	0.9	
			Coliforms	0.6	
1%	Sausage	Chitosan coating	Yeasts and moulds	0.6–1.5	[71]
Turmeric—2% *w*/*w*	Chicken	Carboxymethyl cellulose (CMC) coating	Mesophilic bacteria	2.2–3	[68]
			Psychrotrophic bacteria	3.5	

The synergistic effect of anti-microbial agents or their component, where it has been studied, is not cited. ^1^ The difference of population that is cited is sometimes an approximate number, because of lack of exact data; ^2^ When the range of population difference is cited; it corresponds to the different experimental conditions studied (e.g., different concentrations, storage temperatures, etc.); ^3^ When the anti-microbial agents were combined with other preservation methods or were incorporated into chitosan coatings, the effect of the agent alone is cited; ^4^ These classifications apply to the end point of each experiment; ^5^ The population difference cited corresponds to the 4th day of the experiment, because of lack of data for the pork packed with the fresh-keeping film; ^6^ The population difference cited corresponds to the 4th day of the experiment, because of lack of data for the control sample of ground chicken; ^7^ The population difference cited corresponds to the 10th day of the experiment, because of lack of data for the beef stored at aerobic conditions.

**Table 3:** Overview of studies testing the efficacy of flavonoid anti-microbial agents when added to meat products or incorporated into packaging materials.

Anti-Microbial Agent—Concentration Applied	Food Product	Packaging Material	Microorganisms Targeted	Indicative Reduction (Log_10_ CFU/g, Log_10_ CFU/mL, Log_10_ CFU/cm^2^) ^1,2,3,4^	Reference
Bee pollen—20% *v*/*v*	Salami	Polypropylene (PP) film coated with chitosan solution	*L. monocytogenes*	0.2–1.5	[137]
Cranberry—2.5%, 5%, 7.5% *w*/*w*	Ground beef	-	Total viable bacteria	1.5–2.7	[138]
			*E. coli* O157:H7	0.4–2.4	
2%	Pork slurry	-	*L. monocytogenes*	4.45	[139]
			*Pseudomonas putida*	3.4–3.64	
			*B. thermosphacta*	5.4–7	
			Aerobic mesophilic bacteria	3–3.5	
2%	Pork burger	-	*L. monocytogenes*	3.78	[139]
			*P. putida*	3.5–4	
			*B. thermosphacta*	4	
			Aerobic mesophilic bacteria	1.5	
			Lactic acid bacteria	1.4	
2%	Cooked ham	-	*L. monocytogenes*	1.6	[139]
			Aerobic mesophilic bacteria	2.3–7.3	
			Lactic acid bacteria	1.7–4.5	
2.5% *w*/*w*	Minced pork	-	*S. aureus*	>3.7	[140]
			*L. monocytogenes*	>5	
			*E. coli* O26	>3.32	
			S. Enteritidis	>3.54	
Grape seed extract—5%, 10%, 15% *w*/*w*	Chicken	Chitosan film	Mesophilic bacteria	1–1.5	[141]
			Coliforms	1–2.5	
1200 ppm	Turkey frankfurters	Soy protein coating	*L. monocytogenes*	0	[142]
Grapefruit seed extract—0.5%, 1%	Ground beef	Low density polyethylene (LDPE) film	Total aerobic bacteria	0.5–3	[143]
			Coliforms	0.6	
0.08%	Pork	*Gelidium corneum*–gelatin blend film	*E. coli* O157:H7	0.6	[144]
			*L. monocytogenes*	1.33	
Green tea extract—2.8%	Pork	*Gelidium corneum*–gelatin blend film	*E. coli* O157:H7	1	[144]
			*L. monocytogenes*	0.98	
1200 ppm	Turkey frankfurters	Soy protein coating	*L. monocytogenes*	0	[142]
5%	Hamburger patties	-	Total mesophilic aerobic bacteria	0.2	[145]
			Coliforms	0.55	
			Yeasts and moulds	0.23	
0.5%	Hamburger patties	Chitosan coating	Total mesophilic aerobic bacteria	0.07–0.14	[145]
			Coliforms	0–0.10	
			Yeasts and moulds	0–0.23	
Propolis extract—20% *v*/*v*	Salami	Polypropylene (PP) film coated with chitosan solution	*L. monocytogenes*	2.4–4	[137]
2% *w*/*w*	Beef patties	-	Mesophilic bacteria	0.6–1	[146]
			Psychrotrophic bacteria	0.6–2.6	
0.6% *v*/*w*	Sausage	-	Proteolytic count	2.92 ^5^	[147]
			Lipolytic count	3.23 ^5^	
			Yeasts and moulds	3.13 ^5^	

The synergistic effect of anti-microbial agents or their component, where it has been studied, is not cited. ^1^ The difference of population that is cited is sometimes an approximate number, because of lack of exact data; ^2^ When the range of population difference is cited; it corresponds to the different experimental conditions studied (e.g., different concentrations, storage temperatures, etc.); ^3^ When the anti-microbial agents were combined with other preservation methods or were incorporated into chitosan coatings, the effect of the agent alone is cited; ^4^ These classifications apply to the end point of each experiment; ^5^ The population difference cited corresponds to the 12th day of the experiment, because of lack of data for the control sample.

**Table 4:** Overview of studies testing the efficacy of anti-microbial packaging with bacteriocins on meat products.

Anti-Microbial Agent—Concentration Applied	Food Product	Packaging Material	Microorganisms Targeted	Indicative Reduction (Log_10_ CFU/g, Log_10_ CFU/mL, Log_10_ CFU/cm^2^) ^1,2,3,4^	Reference
Nisin—0.112 g/mL and 1 mL solution in 21 × 38 cm^2^	Beef	High density polyethylene (HDPE) film	Total viable counts	0.5	[187]
			*B. thermosphacta*	2.68	
			Lactic acid bacteria	3.88	
			*Enterobacteriaceae*	0	
			*Pseudomonas* spp.	0	
0.012 g/mL and 4 mL solution in 200 × 300 mm	Beef	Low density polyethylene (LDPE) bags	Total viable counts	0	[188]
			Lactic acid bacteria	2.18	
			*B. thermosphacta*	0.5	
			*Carnobacterium* spp.	0.54	
			*Enterobacteriaceae*	0	
			*Pseudomonas* spp.	0	
0.1125 g/mL and 4 mL solution in 200 × 300 mm	Beef	Low density polyethylene (LDPE) bags	Total viable counts	1.75	[189]
			Lactic acid bacteria	3.45	
			*B. thermosphacta*	2.43	
			*Carnobacterium* spp.	1.78	
			*Enterobacteriaceae*	2.10	
10,000 ppm	Sausage	Collagen casing	Total viable counts	0.2–2	[190]
			*L. monocytogenes*	1–3.2	
0.1125 g/mL and 4 mL solution in 300 × 300 mm	Beef burger	Low density polyethylene (LDPE) bags	Total aerobic bacteria	0.1–2.31	[191]
			Lactic acid bacteria	0–1.24	
			*Staphylococcaceae*	0–0.14	
			*Enterobacteriaceae*	0–0.13	
			Moulds	0	
			Yeasts	0.34	
2000 IU/mL	Chicken	Sodium alginate (NaAlg) coating	Total viable counts	1	[54]
			Psychrotrophic bacteria	0.5	
			*Pseudomonas* spp.	0.7	
			*Enterobacteriaceae*	0.4	
			Lactic acid bacteria	1.8	
			Yeasts and moulds	0.4	
			*L. monocytogenes*	1.4	
Pediocin—25%, 50% *w*/*w*	Ham	Cellulose film	*Listeria innocua*	0.8–2	[192]
			*Salmonella* sp.	0.2–0.5	
Sakasin-A—0.63 mg/cm^2^	RTE Beef carpaccio	Polyethylene (PE)-coated paper sheets	Total aerobic count	0.63	[193]
			*L. innocua*	1.41	

The synergistic effect of anti-microbial agents or their component, where it has been studied, is not cited. ^1^ The difference of population that is cited is sometimes an approximate number, because of lack of exact data; ^2^ When the range of population difference is cited; it corresponds to the different experimental conditions studied (e.g., different concentrations, storage temperatures, etc.); ^3^ When the anti-microbial agents were combined with other preservation methods, the effect of the agent alone is cited; ^4^ These classifications apply to the end point of each experiment.
